# Predictors and influence of postoperative moderate-to-severe pain of PACU in the patients with malignancy

**DOI:** 10.1186/s12871-024-02464-2

**Published:** 2024-02-27

**Authors:** Yu Zhang, Qinxue Dai, Kaiwei Xu, Haifeng Fu, Anqi Zhang, Wenwen Du

**Affiliations:** Departments of Anesthesiology, The First Affiliated Hospital of Wenzhou Medical University, Ouhai District, Wenzhou, Zhejiang P.R. China

**Keywords:** Cancer, Radical surgery, Moderate to severe pain, Epidural analgesia, Vomiting

## Abstract

**Background:**

This study was identified the risk factors for and designed to investigate influence of postoperative moderate-to-severe pain of post anaesthesia care unit (PACU) in patients with malignancy.

**Methods:**

A retrospective study was performed on 22,600 cancer patients with malignancy who underwent elective radical surgery in the new hospital of First Affiliated Hospital of Wenzhou Medical University, between January 2016 and June 2021. All patients were transferred to the PACU after tracheal extubation. Patients were divided into two groups according to a visual analogue scale (VAS) score of > 3: the no-moderate-severe-pain group and moderate-to-severe-pain group. Data pertaining to demographic, surgical, anaesthetic, and other factors were recorded. Lasso and logistic regression analysis was performed to explore the risk factors, then a nomogram was constructed to predict the moderate-severe-pain in the PACU. Validation was performed by using another 662 cancer patients in old hospital. The ROC curves and calibration curve were used to evaluate the accuracy and predictive ability of the nomogram.

**Results:**

The incidence of postoperative moderate-to-severe pain of PACU in patients with malignancy was 1.42%. Gender, type of surgery, postoperative use of PCA, intraoperative adjuvant opioid agonists, NSAIDS, epidural analgesia, duration of anaesthesia, intraoperative massive haemorrhage, PACU vomiting were independent predictors for postoperative moderate-to-severe pain of PACU in the patients with malignancy. The area under the ROC curve of the predictive models in the primary and validation groups were 0.817 and 0.786, respectively. Moderate-to-severe pain in the PACU correlated with hypertension, hyperglycaemia, agitation, and hypoxemia (*P* < 0.05).

**Conclusions:**

The prediction model for postoperative moderate-to-severe pain of PACU in patients with malignancy has good predictive ability and high accuracy, which is helpful for PACU medical staff to identify and prevent postoperative moderate-to-severe pain in advance.

**Trial registration:**

The study was approved by the Clinical Research Ethics Committee of the First Affiliated Hospital of Wenzhou Medical University (No.KY2021-097) and registered in the Chictr.org.cn registration system on 06/12/2021 (ChiCTR2100054013).

## Background

The incidence of cancer has been increasing annually since 2008 in China [1]. According to statistics, approximately 4.82 million new cancer cases will be reported in 2022 [2]. Surgery is the most basic and effective method for treating tumour diseases. Acute postoperative pain is one of the most common complications of radical surgery and refers to acute traumatic pain after surgery, usually at ≤ 7 days. According to an American survey study, 86% of patients experience pain after surgery, and 75% experience moderate-to-severe pain immediately after surgery [3]. Previous studies have shown that approximately 7.3–26.9% of patients in the post anaesthesia care unit (PACU) experience pain [4, 5]. Although we use local anaesthetics, opioids, cyclooxygenase inhibitors, derivatives of herbal preparations, and physical intervention to prevent and treat postoperative pain, acute postoperative pain remains a major clinical problem [6, 7]. 

Acute postoperative pain can cause complications, such as agitation, hypertension, hyperglycaemia, atelectasis, incision splitting, bleeding, and so on [8]. Furthermore, myocardial ischemia, heart failure, and cerebrovascular accidents may also occur [9]. Therefore, the risk factors of acute postoperative pain and how to intervene in advance of acute postoperative pain have become key points of accelerated rehabilitation surgery.

Previous studies have shown that age, body mass index (BMI), preoperative chronic pain, anxiety, and type of surgery may be associated with acute postoperative pain [10, 11]. However, there is still a lack of a common scale for predicting moderate-to-severe pain. Studies on acute pain in the PACU are relatively insufficient. Therefore, we designed a large sample study to retrospectively investigate the incidence of and identify the risk factors of postoperative moderate-to-severe pain of PACU in patients with malignancy.

## Methods

### Study design and participants

This was a single-centre retrospective study. The study was conducted in accordance with the Declaration of Helsinki, and approved by the Clinical Research Ethics Committee of the First Affiliated Hospital of Wenzhou Medical University (No.KY2021-097), and registered in the Chictr.org.cn registration system on 06/12/2021 (ChiCTR2100054013). This study included 22,600 patients with cancer who underwent elective radical surgery in the new hospital area of the First Affiliated Hospital of Wenzhou Medical University, between January 2016 and June 2021. Validation was performed by using another 662 cancer patients in old hospital area. Oral informed consent was obtained from all subjects involved in the study.

The inclusion criteria were scheduled for elective radical surgery, patients with malignant tumour, tracheal intubation general anaesthesia, and transfer to the PACU after tracheal extubation. Anaesthesia records and recovery room record sheets were available.

Individuals with benign tumours intraoperatively, emergency surgery, patients transferred to the intensive care unit (ICU) after radical surgery, unplanned re-entry to the operating room for surgery, multiple metastatic cancers and mental illness were excluded.

### Main outcome measurements

The primary outcome was the incidence and severity of acute moderate-to-severe pain in the PACU. Pain intensity was assessed using the visual analogue scale (VAS). The use of analgesics was also recorded. Patients were divided into two groups according to a VAS score of > 3: the no moderate-severe-pain group and moderate-to-severe-pain group.

### Collection of variables

Age, sex, American Society of Anesthesiologists (ASA) grade, operation mode, type of operation, intraoperative nerve block, intraoperative medication (auxiliary use non-steroidal anti-inflammatory drugs (NSAIDs), opioids, Dexmedetomidine, etc.), operation duration, use of patient-controlled anaesthesia (PCA), intraoperative bleeding, hypothermia, and PACU complications (vomiting, incision bleeding, agitation, hypoxemia, etc.) were collected using medical record systems. The Ramsay sedation score used for sedation assessment.

### Definitions

Hypothermia was defined as a first ear temperature of < 36 °C immediately after admission to the PACU. Vomiting was defined as the expulsion of gastric contents through the mouth in the PACU. Hypoxemia was defined as desaturation < 90% and the performance of one or more appropriate interventions to improve saturation (e.g., tactile stimulation, airway repositioning, oxygen administration, increased oxygen, and positive pressure ventilation) [12]. Intraoperative massive haemorrhage was defined as blood loss > 1000 ml [13]. Hypertension was defined as a blood pressure increase of 30% from the baseline or the use of antihypertensive drugs. Hyperglycaemia was defined as a blood glucose > 11.1 mmol/L. Agitation was defined as a Richmond Agitation Sedation Scale (RASS) score (Supplementary Appendix) of + 3 or + 4 during the PACU stay [14]. 

### Statistical analysis

Statistical analysis was performed using R 4.3.1 software. Continuous variables were summarized as median and interquartile range or mean and standard deviation, as appropriate. Categorical variables are summarized as frequencies and percentages. The chi-square test was used to compare proportions, and the independent sample t-test or Wilcoxon rank-sum test was used to compare continuous variables. Univariate, multivariate and step logistic regression analyses were conducted to determine the risk factors of postoperative moderate-to-severe pain of PACU in the cancer patients.

In the univariate logistic regression analyses, 13 variables were significantly related to moderate-to-severe pain of PACU. Then these 13 variables were considered as potential predictors. But logistic regression analysis often has collinear interference. We applied a LASSO regression algorithm based on each variable for variables selection. After univariate logistic regression analysis, we used LASSO regression analyses to determine the optimal weighting coefficients of all variables. Then 12 retained variables were used for stepwise logistic regression analysis.

We constructed a nomogram of the stepwise logistic regression analysis. Moreover, to verify the nomogram models externally, a total of 662 cancer patients as the external validation datasets from the old hospital of the First Affiliated Hospital of Wenzhou Medical University were included in our study. The receiver operating characteristic (ROC) curves and calibration curve were used to evaluate the accuracy and predictive ability of the nomogram in the external validation datasets. All statistical tests were two-tailed, and a *P* < 0.05 was considered to be statistically significant.

## Results

A total of 22,600 cancer patients who underwent elective radical surgery were included in the new hospital, of whom 316 (1.40%) had moderate-to-severe pain in the PACU. The pain score in the PACU was 1.05 ± 0.42. There were 10,805 males (47.81%) and 11,795 females (52.19%), aged 58.76 ± 13.28 years.

### Comparison of the baseline clinical characteristics between no moderate-to-severe-pain group and moderate-to-severe-pain group in the PACU.

Age, gender, ASA classification, type of surgery, intraoperative massive haemorrhage, intraoperative use of NSAIDs, dexmedetomidine and Opioid agonists, duration of surgery, duration of anaesthesia, intraoperative epidural analgesia, postoperative vomiting, and hypothermia between No moderate-to-severe-pain group and Moderate-to-severe-pain group were significantly different (*P* < 0.05) (Table 1).

**Table 1:** Comparison of the baseline clinical characteristics between No moderate-to-severe-pain group and Moderate-to-severe-pain group in the PACU

Variables	No moderate-to-severe-pain	Moderate-to-severe-pain	P Value
Number	22,284	316	
Age, mean ± SD	58.70 ± 13.30	63.03 ± 11.06	<0.001
Gender, n(%)			<0.001
Male	10,576(47.5%)	229(72.5%)	
Female	11,708(52.5%)	87(27.5%)	
ASA classification, n(%)			0.027
I	2150(9.6%)	27(8.5%)	
II	18,020(80.9%)	245(77.5%)	
III	2114(9.5%)	44(13.9%)	
Preoperative pain, n(%)	3823 (17.2%)	63 (19.9%)	0.190
Type of surgery, n(%)			<0.001
Head or face surgery	1596(7.2%)	8(2.5%)	
Thyroid or breast surgery	3313(14.9%)	9(2.8%)	
Thoracic surgery	4890(21.9%)	67(21.2%)	
Abdominal surgery	12,362(55.5%)	231(73.1%)	
Limb surgery	123(0.6%)	1(0.3%)	
Postoperative use of PCA, n(%)	12,767(57.3%)	233(73.7%)	<0.001
Intraoperative adjuvant medication, n(%)			
Dexmedetomidine	5271(23.7%)	96(30.4%)	0.005
Opioid Agonists	19,546(87.7%)	261(82.6%)	0.006
Opioid Agonists and Antagonists	7610(34.2%)	101(32.0%)	0.42
NSAIDS	12,770(57.3%)	125(39.6%)	<0.001
Epidural analgesia, n(%)	6944 (31.2%)	25 (7.9%)	<0.001
Regional block analgesia, n(%)	658(3.0%)	6(1.9%)	0.27
Duration of anaesthesia	172.38 ± 98.30	213.57 ± 91.49	<0.001
Intraoperative massive haemorrhage, n(%)	529(2.4%)	19(6.0%)	<0.001
PACU vomiting, n(%)	306(1.4%)	40(12.7%)	<0.001
Hypothermia, n(%)	2723(12.2%)	70(22.2%)	<0.001

Univariate logistic regression analyses was conducted to determine the risk factors of postoperative moderate-to-severe pain of PACU in the cancer patients. 13 variables were significantly related to moderate-to-severe pain of PACU and considered as potential predictors (Table 2).

**Table 2:** The results of Univariate, Multivariable and Stepwise logistic regression analysis

Variables	Univariate		Multivariable		Stepwise	
	OR(95%CI)	P	OR(95%CI)	P	OR(95%CI)	P
Age	1.03(1.02–1.04)	< 0.001	1.001(0.99–1.01)	0.920		
Gender						
Male	1(reference)		1(reference)		1(reference)	
Female	0.34(0.27–0.44)	< 0.001	0.45(0.34–0.58)	< 0.001	0.45(0.34–0.58)	< 0.001
ASA classification						
I	1(reference)					
II	1.08(0.73–1.61)	0.697				
III	1.66(1.02–2.69)	0.040				
Type of surgery						
Head and Neck Surgery	1(reference)		1(reference)		1(reference)	
Thyroid or breast surgery	0.54(0.21–1.41)	0.208	1.63(0.60–4.47)	0.339	1.62(0.59–4.52)	0.348
Thoracic surgery	2.73(1.31–5.70)	0.007	4.60(2.04–10.37)	< 0.001	4.60(2.15–11.10)	< 0.001
Abdominal surgery	3.72(1.84–7.56)	< 0.001	8.99(4.20-19.23)	< 0.001	9.02(4.50-20.78)	< 0.001
Limb surgery	1.62(0.20-13.07)	0.650	3.14(0.38–26.18)	0.290	3.11(0.16–17.94)	0.294
Postoperative use of PCA	2.09(1.63–2.69)	< 0.001	1.51(1.12–2.05)	0.006	1.47(1.11–1.97)	0.007
Intraoperative adjuvant medication						
Dexmedetomidine	1.41(1.11–1.79)	0.004	0.92(0.70–1.20)	0.528		
Opioid agonists	1.50(1.21–2.02)	0.006	1.93(1.39–2.68)	< 0.001	1.95(1.39–2.68)	< 0.001
NSAIDS	0.49(0.39–0.61)	< 0.001	0.46(0.36–0.58)	< 0.001	0.46(0.37–0.59)	< 0.001
Epidural analgesia	0.19(0.13–0.29)	< 0.001	0.07(0.05–0.11)	< 0.001	0.07(0.05–0.11)	< 0.001
Duration of anaesthesia	1.003(1.003–1.004)	< 0.001	1.003(1.001–1.004)	< 0.001	1.003(1.001–1.004)	< 0.001
Intraoperative massive haemorrhage	2.69(1.68–4.32)	< 0.001	2.49(1.50–4.14)	0.001	2.51(1.47–4.07)	< 0.001

### Variable selection by LASSO regression analyses

**Fig. 1:**
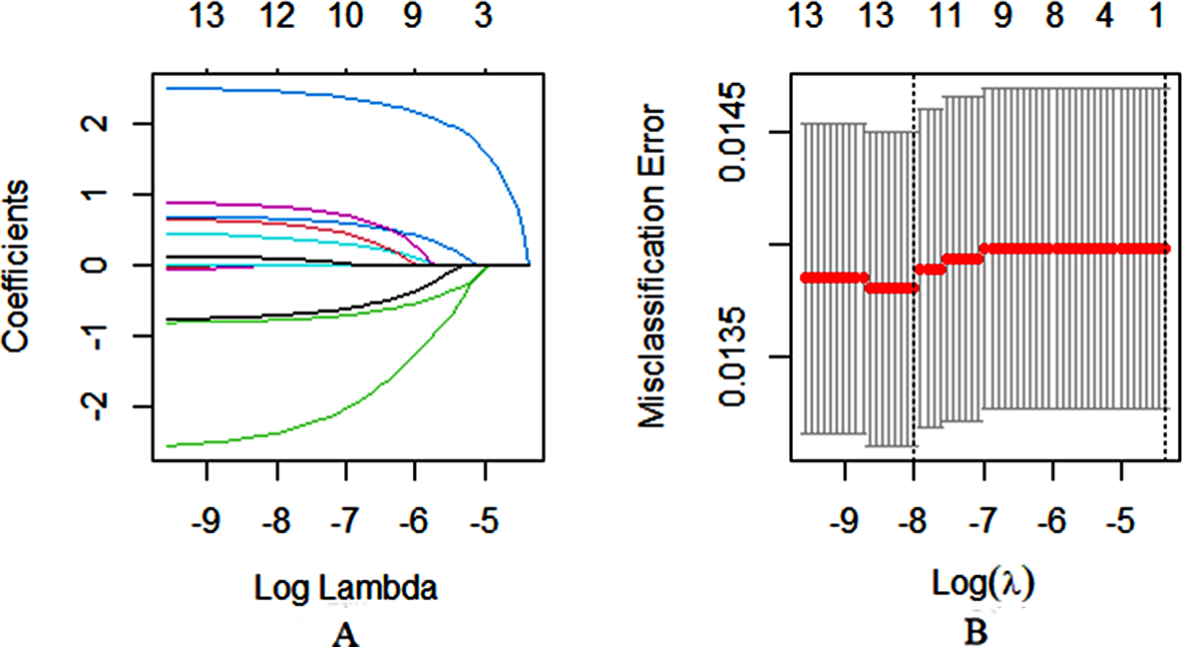
Variable selection by LASSO regression analyses

We applied a LASSO regression algorithm based on each variable for variables selection. In the LASSO analysis, the most appropriate tuning parameter was 0.013 when the partial likelihood binomial deviance reached its minimum value (Fig. 1B). 12 variables (excluding ASA classification) with nonzero coefficients were retained in the LASSO analysis (Fig. 1A). The 12 retained variables were used for multivariate and stepwise logistic regression analysis.

### Multivariate and stepwise logistic regression analyses

The results of stepwise logistic regression analysis showed that gender, type of surgery, postoperative use of PCA, intraoperative adjuvant opioid agonists, NSAIDS, epidural analgesia, duration of anaesthesia, intraoperative massive haemorrhage, PACU vomiting were independent predictors for postoperative moderate-to-severe pain of PACU in the patients with malignancy.(As shown in Table 2).

### Development and validation of a nomogram

**Fig. 2:**
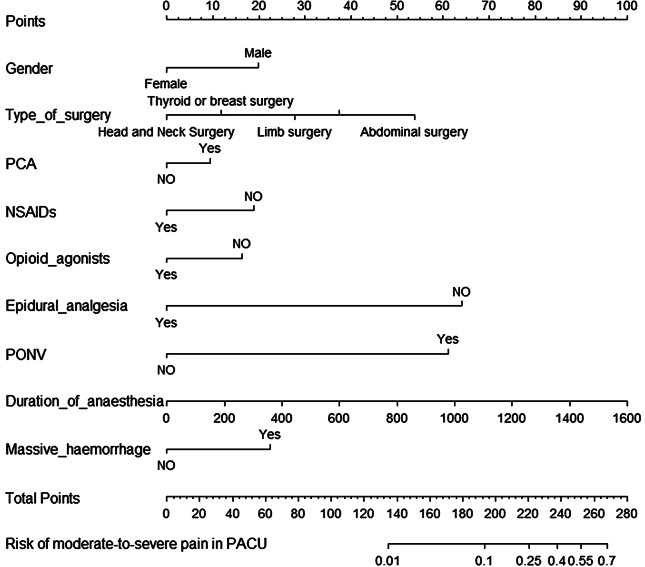
Nomogram of the stepwise logistic regression analysis

**Fig. 3:**
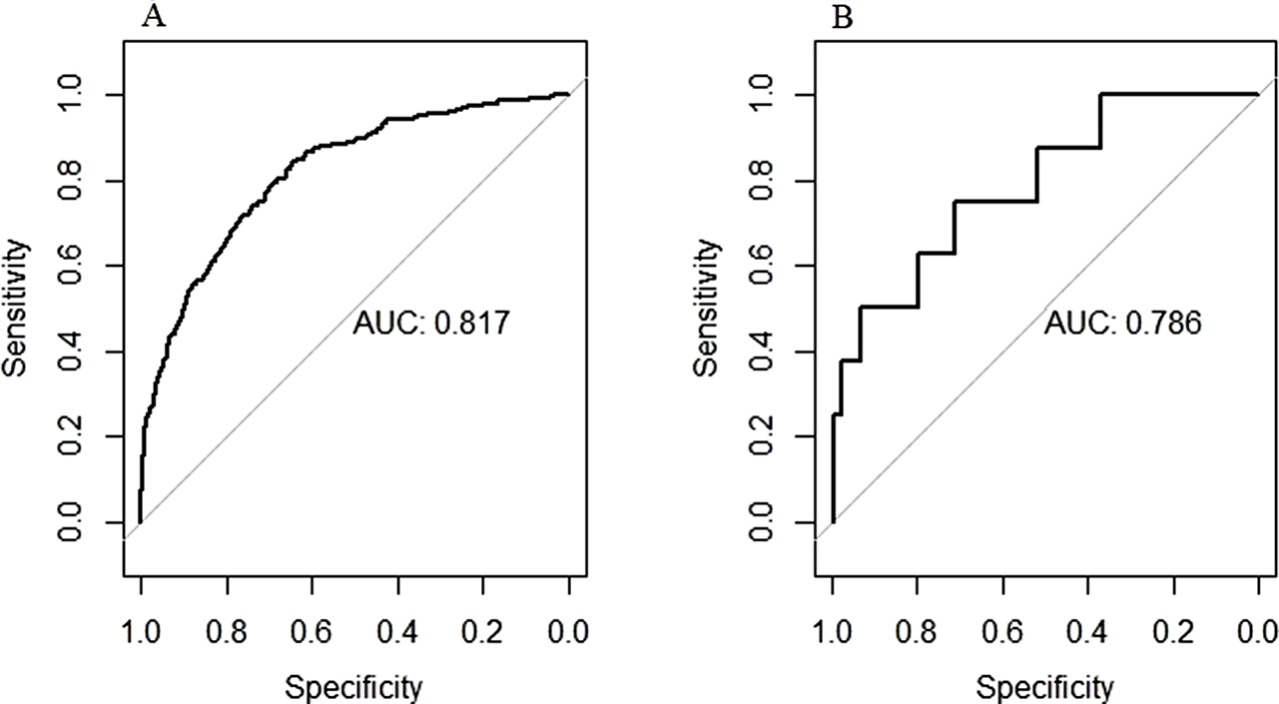
Receiver operating characteristic curves of the nomogram model in the primary and validation groups

**Fig. 4:**
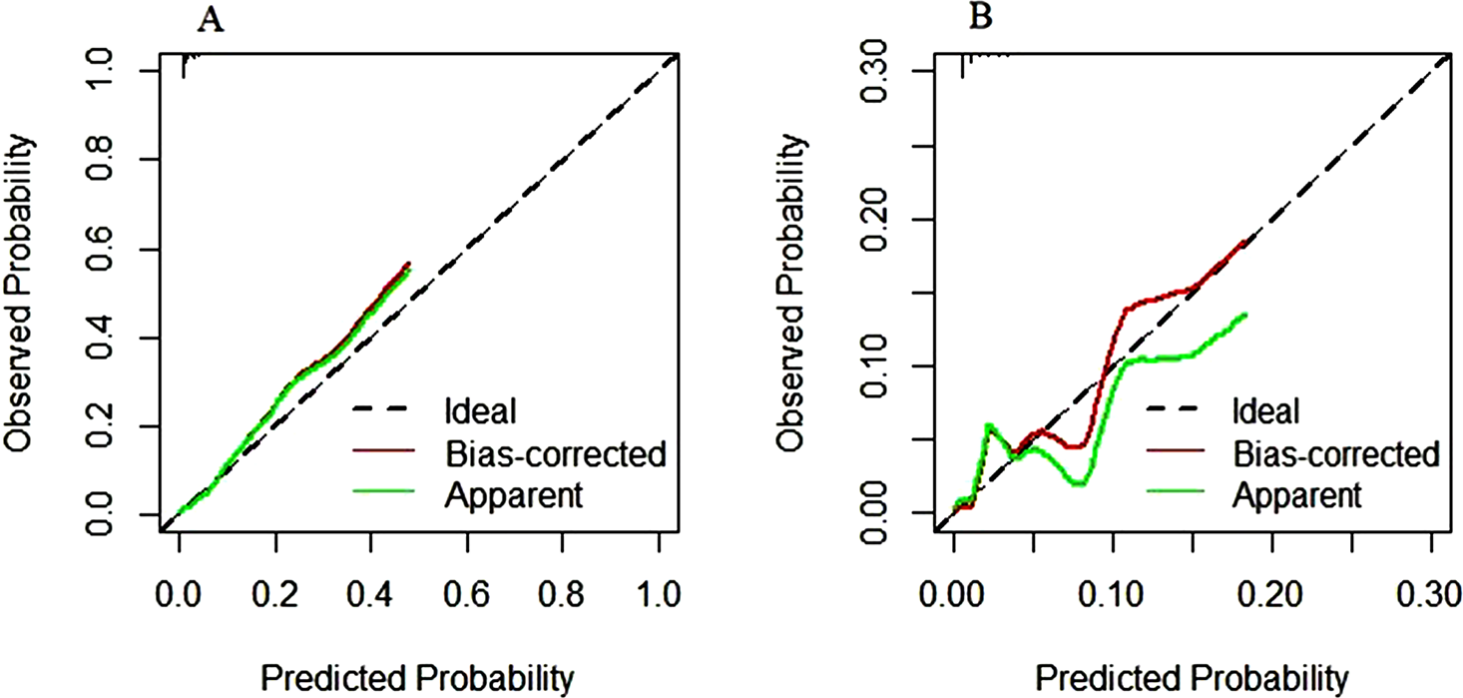
Calibration curves of the nomogram model in the primary and validation groups

The nomogram was constructed after stepwise logistic regression analysis to predict the probability of postoperative moderate-to-severe pain of PACU in the patients with malignancy (Fig. 2). Validation was performed by using another 662 cancer patients. The ROC curves and calibration curves were used to evaluate the accuracy and predictive ability of the nomogram model (Figs. 3 and 4). The area under the curve (AUC) of the predictive model in the primary and validation groups were 0.817 (Fig. 3A) and 0.786 (Fig. 3B), respectively.

### Effect of moderate-to-severe pain in the PACU

The study found that moderate-to-severe pain in the PACU significantly increased the incidence of hypertension, hyperglycaemia, Agitation, and hypoxemia in the recovery room (*P* < 0.05)(Table 3).

**Table 3:** Effects of moderate to severe pain in the PACU on patient recovery

Variables	Non-moderate to severe pain	Moderate to severe pain	P Value
Hypertension, n(%)	463(2.1%)	17(5.4%)	0.02
Hyperglycaemia, n(%)	254(1.1%)	8(2.5%)	<0.001
Agitation, n(%)	22 (1.0%)	29(9.2%)	<0.001
Hypoxemia, n(%)	5014(22.5%)	132(41.8%)	<0.001

## Discussion

A total of 22,600 patients with cancer after elective radical surgery were included in this study, and the incidence of moderate-to-severe pain in the PACU was 1.42%. Gender, type of surgery, postoperative use of PCA, intraoperative adjuvant opioid agonists, NSAIDS, epid ural analgesia, duration of anaesthesia, intraoperative massive haemorrhage, PACU vomiting were independent predictors for postoperative moderate-to-severe pain of PACU in the patients with malignancy. Female sex, intraoperative use of NSAIDs, opioid agonists and epidural analgesia were protective factors for moderate-to-severe pain in the PACU. Moderate-to-severe pain in the PACU is associated with hypertension, hyperglycaemia, agitation, and hypoxemia.

The AUC of the predictive models in the primary and validation groups were 0.817 and 0.786, respectively. The prediction model for postoperative moderate-to-severe pain of PACU in patients with malignancy has good predictive ability and high accuracy, which is helpful for PACU medical staff to identify and prevent postoperative moderate-to-severe pain in advance.

Previous studies have shown that approximately 7.3–26.9% of patients in the post anaesthesia care unit (PACU) experience pain [4, 5]. The incidence of moderate-to-severe pain in the PACU was 1.42% in our study. The reasons for this are as follows. First, intraoperative epidural analgesia are effective in the reduction of pain and stay in the PACU. In our study, 6969(30.84%) patients underwent epidural analgesia during surgery. Second, most patients routinely use patient-controlled analgesia in the study. Finally, all the patients were admitted to the PACU at our hospital after extubation. The patient will only stay in the recovery room for 1 h unless special circumstances occur. Therefore, the incidence of postoperative pain of PACU in our study was relatively low.

There were sex-related differences in pain. In contrast to previous studies [15], the incidence of moderate-to-severe pain in the PACU in the male group was higher than that in the female group in this study. Male sex was a risk factor for moderate-to-severe pain in the PACU. Patients with malignant tumours selected in this study required intraoperative indwelling catheters because of the long surgery time. In the PACU, the incidence of agitation due to catheter-related bladder discomfort was significantly higher than that in women [16] (chi-square value = 149.84, *P* < 0.001). Postoperative agitation can result in incision pain in males.

Surgery, as a type of stimulation, can cause local or systemic inflammation, which can lead to pain. Surgery can lead to a breakdown of the integrity of the skin, resulting in exposure of nerve endings in the skin, which can be stimulated to produce ectopic electrical currents that cause pain [17]. This study confirmed that the incidence of moderate-to-severe pain in the PACU was significantly higher in chest and abdominal surgeries.

NSAIDs are the first-line treatment option for most patients with acute mild-to-moderate pain [18]. NSAIDs can not only inhibit prostaglandin synthesis but also inhibit lymphocyte activity and activation of T lymphocyte differentiation and reduce the stimulation of afferent nerve endings. It acts directly on nociceptive receptors and prevents the formation and release of pain-causing substances [19]. NSAIDs are widely used because they are non-addictive. This study found that the intraoperative use of NSAIDs was a protective factor against moderate-to-severe pain in the PACU. However, when the dosage of NSAIDs drugs exceeds a certain level, even additional dosage cannot increase the analgesic effect, so it should be used appropriately and reasonably.

Previous studies have found that the incidence of postoperative pain is higher when the operation time exceeds 3 h [20]. This study also confirmed that the duration of anaesthesia was an independent risk factor for moderate-to-severe pain in the PACU. A longer operation time may result in severe tissue damage at the surgical site, increased release of inflammatory mediators, decreased threshold of nociceptors controlling inflammatory tissues, and an enhanced response to normal sensory conduction, resulting in peripheral sensitization. Simultaneously, the release of excitatory amino acids from primary neurons activates excitatory amino acid receptors in the spinal cord, resulting in a high response to nociceptive afferent stimuli (central sensitization).

PONV and postoperative pain are common complications in the PACU. There was a significant association between these two complications [21]. A study noted that nausea was frequently accompanied by pain in the first few hours after surgery. PONV can cause incision dehiscence, increase abdominal muscle tension, aggravating abdominal pain and discomfort [22]. Postoperative vomiting will increase abdominal muscle tension, aggravating abdominal pain and discomfort. So PACU vomiting was considered as a possible predictive factor for analysis. This study showed that the incidence of moderate-to-severe pain in the PACU in patients with postoperative vomiting was significantly higher than that in patients without vomiting.

The incidence of moderate-to-severe pain in the PACU in patients with intraoperative massive haemorrhage is higher than that in patients without massive haemorrhage, which may be caused by the reduction of haemoglobin during massive haemorrhage, resulting in hypotension, hypoperfusion, and lactic acid accumulation [23], thus causing postoperative pain.

This study found that hypertension, hyperglycemia, agitation, and hypoxemia are affected by moderate to severe pain. Pain can stimulate the sympathetic nervous system and the adrenal glands to release adrenaline [24]. This increases the heart rate and constricts blood vessels which increases blood pressure and hyperglycemia. In addition, pain can acutely induce the release of catecholamines and a short-term pro-inflammatory sympathetic response [25], which may lead to postoperative agitation. Patients with pain tend to hold their breath during a flare-up of pain or breathe fast and shallow.

Previous studies have shown that preoperative anxiety are significantly associated with postoperative acute pain. However, this was a single-centre, retrospective study; therefore, complete data were missing. However, this study explored the risk factors of moderate-to-severe pain in the PACU for patients with cancer after elective radical surgery based on big data, so it is still of high clinical significance.

## Conclusion

Gender, type of surgery, postoperative use of PCA, intraoperative adjuvant opioid agonists, NSAIDS, epidural analgesia, duration of anaesthesia, intraoperative massive haemorrhage, PACU vomiting were independent predictors for postoperative moderate-to-severe pain of PACU in the patients with malignancy. Female sex, intraoperative use of NSAIDs, opioid agonists and epidural analgesia were protective factors for moderate-to-severe pain in the PACU.The area under the ROC curve of the predictive models in the primary and validation groups were 0.817 and 0.786, respectively. The prediction model for postoperative moderate-to-severe pain of PACU in patients with malignancy has good predictive ability and high accuracy, which is helpful for PACU medical staff to identify and prevent postoperative moderate-to-severe pain in advance.

## Data Availability

The datasets used or analysed during the current study are available from the corresponding author on reasonable request. The email address of the corresponding author is 862892574@qq.com.

## Abbreviations

ASA: American Society of Anesthesiologists;
BMI: body mass index;
ICU: intensive care unit;
NSAID: non-steroidal anti-inflammatory drug;
PCA: patient-controlled anaesthesia
PACU: post anaesthesia care unit;
VAS: visual analogue scale
